# Opportunities and Challenges With Creative Community Partnerships

**DOI:** 10.1093/geroni/igaa057.2962

**Published:** 2020-12-16

**Authors:** Melinda Heinz

## Abstract

Arts programming can address chronic conditions prevalent among older adults. An overview of an implementation of the revised nationwide Opening Minds through Arts (OMA) program anchors the symposium. The paper reports an implementation by an area university and an eldercare facility to recruit and train student volunteers to collaborate with persons living with dementia, and create paintings for a public exhibition. The Arts for a Lifetime Program used bi-weekly student led programming in a long-term care community; the paper includes an overview of materials used throughout the program and reports resident preferences for materials. A report of results of an ethnographic investigation of the impact of creative aging fine arts programs on older adults adds information about how participation might influence the older person’s self-esteem or perceptions of aging. The presentation about creation of music modules investigates the potential of music therapy for the promotion of healing for older adults managing pain. The final paper describes the methodology lessons learned from ARTmail, a community engaged study of the benefits of a structured participatory arts program for older adults with memory symptoms or cognitive impairment. Presentations in this symposium inform identification and development of opportunities to create and engage in meaningful experiences with older adults.

